# A study of the aphrodisiac properties of *Cordyceps militaris* in streptozotocin-induced diabetic male rats

**DOI:** 10.14202/vetworld.2021.537-544

**Published:** 2021-02-27

**Authors:** Toan Van Nguyen, Pramote Chumnanpuen, Kongphop Parunyakul, Krittika Srisuksai, Wirasak Fungfuang

**Affiliations:** 1 Department of Agricultural Biotechnology, Faculty of Biotechnology, Ho Chi Minh City Open University, Vietnam; 2 Department of Zoology, Faculty of Science, Kasetsart University, Bangkok 10900, Thailand; 3 Omics Center for Agriculture, Bioresources, Food and Health, Kasetsart University (OmiKU), Bangkok, Thailand

**Keywords:** *Cordyceps militaris*, diabetes mellitus, sexual behavior, sperm, streptozotocin

## Abstract

**Background and Aim:**

*Cordyceps militaris* (CM) is a fungus that has been used to enhance aphrodisiac activity in men, but to date, no studies have focused on its antidiabetic properties. This study aimed to investigate the effects of CM on reproductive performance of streptozotocin (STZ)-induced diabetic male rats.

**Materials and Methods:**

Six-week-old Wistar rats were randomly divided into four groups: control Group 1 consisting of healthy rats; Group 2, healthy rats treated with CM (100 mg/kg); Group 3, diabetic untreated rats; and Group 4, diabetic rats treated with CM (100 mg/kg). Rats were orally administered with vehicle or CM for 21 days. The body weight, blood glucose level, food intake, epididymal sperm parameter, sexual behavior, serum testosterone level, and antioxidant parameters were determined.

**Results:**

The results indicated that CM treatment in STZ-induced diabetic rats significantly improved the epididymal sperm parameter and serum testosterone level and, in turn, their copulatory behavior. CM treatment in diabetic rats significantly ameliorated malondialdehyde level and significantly improved the glutathione and catalase levels.

**Conclusion:**

These results provide new information on the pharmacological properties of CM in ameliorating testicular damage due to oxidative stress and improving sexual performance in diabetic male rats.

## Introduction

Diabetes mellitus (DM) is a metabolic disorder characterized by chronic hyperglycemia, which often results from deficiency in insulin secretion or action [1,2]. The number of patients with DM has been rapidly increasing in both children and adults [3,4]. DM also increases the risk of developing other related complications, such as atherosclerosis, peripheral vascular disease, hypertension, retinopathy, neuropathy, and reproductive problems [5,6]. DM has often been associated with sexual dysfunction in men and women. The previous studies indicate that DM adversely affects male fertility in both humans and animals [7-10]. Male patients with DM have sexual dysfunctions, such as decreased libido and impotence, spermatogenesis, steroidogenesis, problems with penile erection and ejaculation, and disturbance in endocrinal hormone secretion (hypothalamic-pituitary-gonadal axis), which can lead to infertility [11,12]. Oxidative stress is an important component of reproductive damage in a patient with DM. The previous studies indicated that DM causes increased lipid peroxidation in the testis and epididymal sperm, can stimulate the production of reactive oxygen species (ROS) [1,13,14], and decreases the catalase (CAT), glutathione (GSH) reductase (GR), and GSH peroxidase (GPx) levels and efficacy of superoxide dismutase (SOD) antioxidant activity [15].

*Cordyceps*, a fungus associated with the phylum Ascomycota, is known as a parasite residing in the Lepidoptera larvae or pupae and includes *Cordyceps sinensis*, *Cordyceps militaris* (CM), and other fungi [16,17]. This fungus has been used as a food tonic and traditional medicine in East and Southeast Asia [18]. It contains many bioactive compounds, such as cordycepin, polysaccharide, cordycepic acid, adenosine, amino acids, and trace elements, including Zn, Mg, Mn, Cd, and Pb [19-21]. The previous studies have confirmed that CM has antitumor, anti-inflammatory, antioxidant, and antibacterial properties [22-24]. In traditional medicine, it is believed that *C. sinensis* and CM have the potential to resolve problems related with infertility, impotency, seminal emission, and sexual impairment [25]. Recently, many researchers have been interested in studying the effects of natural products on male reproductive function and have reported that some products can significantly reverse the damage induced by DM on the reproductive organs of animals [26,27]. Several studies have shown that CM can improve the quality and quantity of sperm in rats and boars [16,28] and prevent bisphenol A (BPA)-induced reproductive damage in rats [20].

However, there are no scientific reports on the effect of CM on reproductive function in streptozotocin (STZ)-induced diabetic male rats. The present study aimed to investigate the effects of CM on the reproductive functions in STZ-induced diabetic male rats and understand the mechanism of CM on the reproductive axis in diabetic complications.

## Materials and Methods

### Ethical approval

All procedures were carried out in accordance with the guidelines for the Care and Use of Laboratory Animals of the National Institutes of Health, USA and were approved by the animal use and care committee of Kasetsart University Research and Development Institute, Kasetsart University, Thailand (ID:ACKU60-SCI-014).

### Study period and location

The study was conducted from July 2018 to September 2019 at Department of Zoology, Faculty of Science, Kasetsart University, Bangkok, Thailand.

### Cultivation of CM and preparation of mycelium powder

The mycelia of CM were obtained from Assoc. Prof. Dr. Wanwipa Vongsangnak, Omics Center for Agriculture, Bioresources, Food and Health, Kasetsart University (OmiKU), Bangkok, Thailand. CM were cultured in peptone yeast extract broth with sucrose (PY sucrose) modified from Yang *et al*. [29] consisting of yeast extract powder (20 g/L), peptone (10 g/L), and sucrose (25 g/L). Flask culture was performed in a 250 mL Erlenmeyer flask containing 100 mL of PYG broth inoculated with 2%(w/v) of the seed culture at 20°C on a rotary shaker incubator at 150 rpm in dark condition for 3 weeks. The whole cultured mycelium and media from each flask were harvested and homogenized using an electric fruit blender at highest speed for 20 min. The homogenized samples were freeze-dried and kept at −20°C until used. To ensure cordycepin content in the crude extract from CM mycelium powder, the high-performance liquid chromatography (HPLC) system was applied for cordycepin quantification process according to Zhou *et al*. [30]. Crude extraction and sample preparation were performed according to Wang *et al*. [31]. Quantitative analysis of cordycepin was conducted by evaluating the peak area relative to a standard curve. Peaks of cordycepin and other compounds in the sample were identified through their retention time.

### Animal and housing conditions

A total of 24 male and 20 female Wistar rats (*Rattus norvegicus*), 6 weeks old and weighing approximately 180-200 g were obtained from the National Laboratory Animal Center, Mahidol University, Thailand. The animals were individually housed in standard polypropylene cages with sawdust bedding, with the relative humidity maintained between 60% and 70% and room temperature between 25 ± 2°C. Periods of lighting were controlled with 12-h light/dark cycles. Rats had free access to food and drinking water *ad libitum* throughout the course of the study. The animals were allowed to acclimatize to the laboratory environment for a week before the initiation of the experiment.

### Induction of DM

Type I DM was induced by a single intraperitoneal injection (i.p.) of STZ and prepared by dissolving in a citrate buffer (0.1 M, pH 4.5) at a dose of 60 mg/kg body weight. The control group was administered with 0.1 M citrate buffer. After 3 days, the blood glucose level was tested using a blood glucose meter (Accu-Chek Active, Roche Diagnostic, Germany). Rats with blood glucose level ≥250 mg/dL were considered diabetic and used in this experiment.

### Experimental design

Rats were randomly divided into four groups, with six animals in each group. Group 1 consisted of the control group comprising healthy rats, who were administered 1% Tween suspension. Group 2 consisted of healthy rats treated with 100 mg/kg CM. Group 3 consisted of diabetic rats treated with 1% Tween suspension, and Group 4 consisted of diabetic rats treated with 100 mg/kg CM. Rats were treated with CM mycelium powder suspended in Tween 80 to prepare a 1% suspension by oral gavage for 21 consecutive days.

### Blood glucose level, body weight, and food intake of animals

The blood glucose levels and body weight of rats in all groups were monitored once a week during the experiment. The fasting blood glucose level was measured using a blood glucose meter (Accu-Chek Active, India). The daily food intake of each rat was measured by weighing the remaining chow, with food spillage accounted for in the intake measurement.

### Copulatory behavior test

The copulatory behavior test was conducted according to the previously set protocol [32]. The tests were conducted in the absence of light, with a 40-W red light bulb used to illuminate the room. Adult female rats were ovariectomized and rendered behaviorally estrous and facilitated mating by sequential injection of β-estradiol 3-benzoate (Sigma-Aldrich, St Louis, MO, USA, 10 μg/0.1 mL sesame oil/rat) and progesterone (Sigma-Aldrich, St Louis, MO, USA, 500 μg/0.1 mL sesame oil/rat) 48 h and 4 h before testing, respectively. Each male rat was placed in a transparent acrylic box with sawdust for 10 min so as to become habituated to the surrounding in the test box. A receptive female rat was placed in the box with the male rat, so they were free to have sexual interaction. During the test, the following sexual parameters were recorded: (a) Mount latency, the time elapsed from the entrance of receptive female rat to the first mount; (b) ejaculation latency, the time from the first intromission to the first ejaculation; (c) post-ejaculatory interval (PEI), the time from ejaculation to the next mount; (d) ejaculation frequency (EF), the number of ejaculations within a span of 30 min; and (e) total mount frequency, the number of mounts within a span of 30 min.

### Sperm collection and analysis

At the end of the experiment, all animals were anesthetized using pentobarbital sodium and sacrificed. The testes epididymis and seminal vesicles were collected and weighed. The epididymides cauda were quickly placed in a Petri dish with 2 mL of normal saline and prewarmed at 37°C. Thereafter, the caudal part of the epididymis was cut into small pieces to collect the spermatozoa. The sperm concentration was analyzed using Neubauer hemocytometer cell counting under a light microscope. To determine the sperm viability and its morphology, we smeared a drop of spermatozoa suspension on a glass slide and then stained it with eosin-nigrosin and air-dried. The stain was examined under a light microscope, with 200 spermatozoa screened. Non-stained cells were considered live sperm, and dead cells were stained orange-red because the stain could pass through the cell membrane. The percentage viability and abnormality were determined thereafter.

### Serum testosterone levels

Blood samples were collected from the posterior vena cava. The serum was obtained by centrifugation of the samples at 2200 g for 15 min at 4°C. The serum was kept at −80°C for hormonal analysis. The serum testosterone level was assayed by an enzyme-linked immunosorbent assay (ELISA) kit (Testosterone ELISA Kit, Abcam, Cambridge, UK). The manufacturer’s protocol was followed to detect the presence of the hormone.

### Antioxidant parameter

Malondialdehyde (MDA) analysis was conducted using the method recommended by Dubovskiy *et al*. [33]. Reduced GSH level was estimated following previous protocols [34]. CAT activity was determined according to the method outlined in Lartillot *et al*. [35].

### Statistical analysis

Representative data were expressed as mean ± standard deviation. Statistical analyses were performed by one-way analysis of variance, followed by Tukey *post hoc* test, using the R Project Statistical Computing package (R Core Team, 2019) [36]. The statistical significance was determined at p<0.05.

## Results

### Total cordycepin level in CM mycelium powder

**Figure-1:**
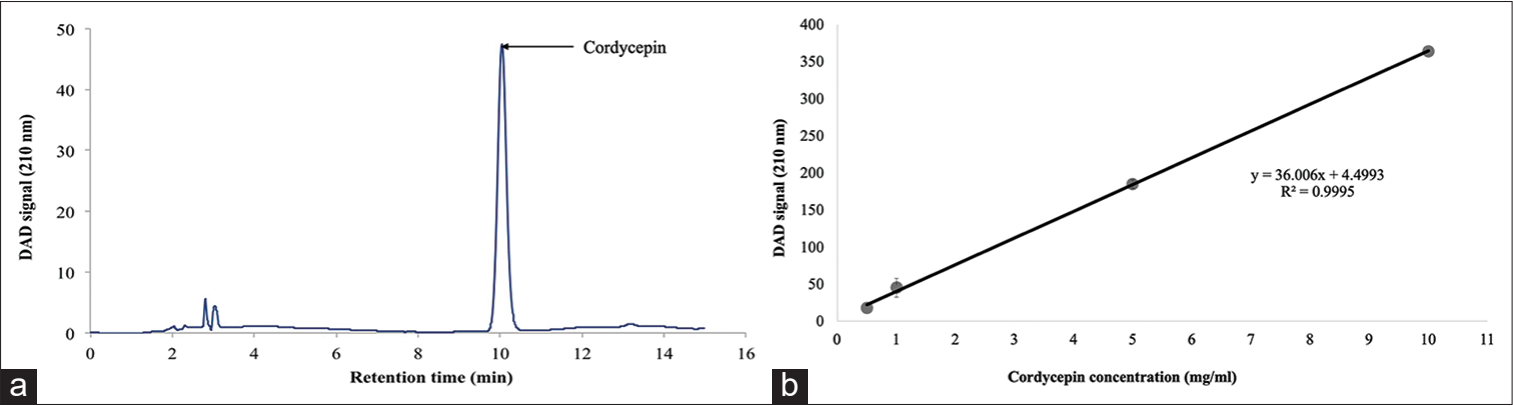
High-performance liquid chromatography analysis of *Cordyceps militaris* (a), standard curve (b).

Cordycepin content in the crude extract from CM mycelium powder was measured by HPLC. Retention time value of standard cordycepin was 10.11 min under an optimal chromatographic condition. The value of a standard sample indicated a linear relation y = 36.006x + 4.4993 (R^2^ = 0.9995). The cordycepin content in mycelium powder was measured in triplicate. The average cordycepin content in mycelium powder was 76.75 mg/g by dry weight (Figure-1A and B).

### Effect of CM on body weight, blood glucose level, and food intake

As shown in Table-1, healthy rats had a significantly higher body weight compared to diabetic rats. STZ-induced diabetic rats had a significantly increased blood glucose level and food intake. CM treatment had no effect on body weight, blood glucose level, and food intake compared to that in diabetic rats. However, CM treatment significantly increased the food intake in diabetic rats compared to healthy rats.

**Table-1:** Effect of CM on body weight, blood glucose level, and food intake.

Groups	Body weight(g)	Blood glucose(mg/dL)	Food intake(g/day)
NC	298.74±30.64^a^	148.33±11.70^a^	15.08±3.26^a^
NC+CM100	273.11±30.07^a^	156.4±23.61^a^	18.03±1.29^a^
DM	167.19±73.01^b^	392±26.87^b^	19.93±6.87^ab^
DM+CM100	144.29±73.29^b^	439.75±99.83^b^	26.91±1.68^b^

All data are shown as the mean±SD.

a,b Superscripts within the same column differ significantly (p<0.05).

NC=Normal control rats; NC+CM100, normal rats treated with *Cordyceps* *militaris* (CM) (100 mg/kg); DM=Diabetic control rats; DM+CM100, diabetic rats treated with CM (100 mg/kg)

### Effect of CM on reproductive organ weight

DM caused a significant decrease in the weight of the epididymis and seminal vesicle. In contrast, CM treatment in diabetic rats indicated a significant decrease in testicular weight compared to that in healthy rats (Table-2).

**Table-2:** The effect of CM on testes, epididymis, and seminal vesicle.

Groups	Reproductive organ weight (g)		

	Testes	Epididymis	Seminal vesicle
NC	3.36±0.21^a^	0.84±0.19^a^	0.66±0.11^a^
NC+CM100	3.12±0.23^a^	0.96±0.17^a^	0.6±0.21^a^
DM	2.81±0.25^ab^	0.53±0.06^ab^	0.16±0.14^b^
DM+CM100	1.84±1.31^b^	0.44±0.39^b^	0.18±0.20^b^

All data are shown as the mean±SD.

a,b Superscripts within the same column differ significantly (p<0.05).

NC=Normal control rats; NC+CM100, normal rats treated with *Cordyceps* *militaris* (CM) (100 mg/kg); DM=Diabetic control rats; DM+CM100, diabetic rats treated with CM (100 mg/kg)

### Effects of CM on the characteristics of epididymal sperm and serum testosterone level

**Figure-2:**
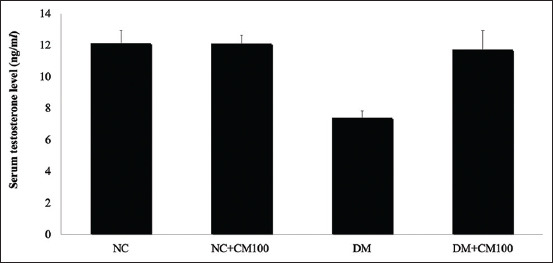
Serum testosterone level. All data are shown as mean±SD. The different characters indicated significant differences (p<0.05). NC=Normal control rats; NC+CM100, normal rats treated with *Cordyceps militaris* (CM) (100 mg/kg); DM=Diabetic control rats; DM+CM100, diabetic rats treated with CM (100 mg/kg). ^a,b^Different letters as superscript indicate the difference between the different groups of experiment.

As shown in Table-3, STZ-induced diabetic rats showed a significant decrease in sperm count, sperm motility, and sperm viability. However, when CM was administered, it improved the epididymal sperm parameters in diabetic rats (p<0.05). The serum testosterone level was significantly lower in STZ-induced diabetic rats, which was significantly increased after treatment with CM (p<0.05; Figure-2).

**Table-3:** Epididymal sperm parameter.

Groups	Epididymal sperm parameter		

	Sperm count(10^6^×/mL)	Sperm motility (%)	Sperm viability (%)
NC	120.50±43.56^a^	88.00±5.70^a^	87.03±4.58^a^
NC+CM100	139.30±55.58^b^	88.60±4.16^a^	87.60±3.05^a^
DM	49.30±26.16^b^	64.30±4.18^b^	77.28±5.22^b^
DM+CM100	93.10±9.27^c^	76.05±4.06^c^	85.01±2.42^a^

All data are shown as the mean±SD.

a Superscripts within the same column differ significantly (p<0.05).

NC=Normal control rats; NC+CM100, normal rats treated with *Cordyceps* *militaris* (CM) (100 mg/kg); DM=Diabetic control rats; DM+CM100, diabetic rats treated with CM (100 mg/kg)

### Effects of CM on copulatory behavior

**Figure-3:**
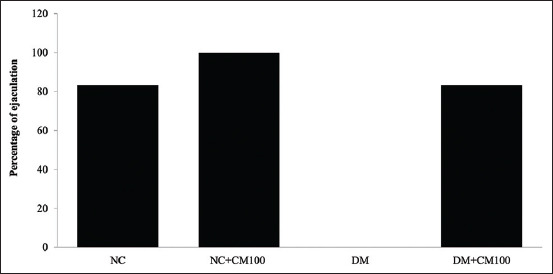
Percentage of ejaculation. NC=Normal control rats; NC+CM100, normal rats treated with *Cordyceps militaris* (CM) (100 mg/kg); DM=Diabetic control rats; DM+CM100, diabetic rats treated with CM (100 mg/kg).

The copulatory behavior is presented in Table-4 and Figure-3. The non-treated diabetic rats showed no copulatory behavior during the sexual test, which showed improvement after CM administration. Interestingly, CM treatment in normal rats reduced the PEI and increased the EF and number of mounts within a 30-min window compared with that in non-treated diabetic rats. CM treatment in both normal and diabetic rats was shown to improve male sexual behavior. The percentage of rats, treated with CM, reaching ejaculation during the copulatory test was 83.33% and 66.67% for healthy and diabetic rats, respectively.

**Table-4:** The effect of CM on copulative behavior.

Groups	Mount latency (min)	Ejaculation latency (min)	Post ejaculatory interval (min)	Ejaculation frequency	TMF30
NC	5.36±2.76	7.75±0.4	0.77±0.11	25.5±0.71	34±1.41
NC+CM100	7.69±1.95	8.18±1.45	0.39±0.04	43.5±2.12	59.5±0.71
DM	N	N	N	N	N
DM+CM100	5.37±1.35	6.01±2.17	1.33±0.23	14±2.83	23.5±2.12

All data are shown as the mean±SD. n=Showing no sexual behavior, NC=Normal control rats; NC+CM100, normal rats treated with *Cordyceps* *militaris* (100 mg/kg); DM=Diabetic control rats; DM+CM100, diabetic rats treated with CM (100 mg/kg)

### Effects of CM on MDA, GSH, and CAT levels

The serum MDA level was significantly higher in the STZ-induced diabetic rats but significantly lower in diabetic rats treated with CM. The antioxidant GSH and CAT levels were significantly lower in diabetic rats compared to those in healthy counterparts (p<0.05). In addition, the GSH levels in CM-treated diabetic rats were similar to those in healthy rats. Therefore, CM administration significantly increased the CAT levels in diabetic rats (p<0.05; Table-5).

**Table-5:** Effect of CM on antioxidant parameters.

Groups	Malondialdehyde (nmol/ml)	Glutathione (umol/L)	Catalase\(U/mL)
NC	36.80±2.28^a^	10.40±0.71^a^	373.51±8.93^a^
NC+CM100	32.8±5.40^a^	10.56±0.75^a^	366.80±10.58^a^
DM	70.30±4.16^b^	6.56±0.76^b^	269.40±10.98^b^
DM+CM100	49.40±2.79^c^	10.68±0.82^a^	318.40±23.65^c^

All data are shown as the mean±SD.

a Superscripts within the same column differ significantly (p<0.05).

NC=Normal control rats; NC+CM100, normal rats treated with *Cordyceps* *militaris* (CM) (100 mg/kg); DM=Diabetic control rats; DM+CM100, diabetic rats treated with CM (100 mg/kg)

## Discussion

The results of the present study demonstrate that DM induced by STZ caused a decrease in the reproductive organ weight, sperm motility, sperm concentration, sperm viability, serum testosterone level, and antioxidant levels and absence of sexual behavior in rats. CM treatment could improve the epididymal sperm parameter, serum testosterone level, antioxidant levels, and sexual behavior in STZ-induced diabetic rats.

In our study, we prepared CM using the whole cultured mycelium and media, without the use of fruiting body. Our results indicate that CM treatment did not affect the body weight and hypoglycemic activity. In addition, the previous studies indicated that the extract of fruiting body or mycelia of CM exhibited antidiabetic activity in DM type 1 and 2 in animal models [37-40]. However, another study indicated that CM mycelial extract has a less potent hypoglycemic activity than fruiting body extract and polysaccharide-enrich fractions [40].

Particularly, a previous study reported an increase in the oxidative stress in STZ-induced diabetic mice in their testis and epididymal sperm during the early diabetic phase by enhancing the MDA and increasing the ROS levels [41]. Our results revealed that STZ-induced DM decreased the epididymal sperm parameter, serum testosterone levels, reproductive organ weight, libido or sexual desire, and fertility within 21 days after STZ administration. Other studies have suggested that DM affects the male reproductive system through overproduction of ROS or oxidative stress and impairment of mitochondrial function [42-44]. Increased ROS seems to affect germ cell apoptosis, impairment of spermatogenesis, sperm morphology, plasma membrane damage, and sperm DNA damage, leading to male infertility [45,46]. Hyperglycemia disrupts the synthesis of adenosine triphosphate and increases the production of ROS in the mitochondria, probably leading to decreased sperm viability and sperm motility, and other functional disorders [47]. In the current study, we observed an increased MDA level in the STZ-induced diabetic rats and decreased GSH and CAT levels, which are in agreement with the previous findings associated with oxidative stress in DM [48,49]. It has been previously reported that cordycepin increased the SOD, CAT, GPx, GR, GST, and GSH activities, while it decreased the lipid peroxidation in aged rats [50]. Other studies indicated that antioxidant supplementation might not be beneficial against DM-induced reproductive dysfunction [1,51-54]. In this study, we observed a significant decrease in the MDA level and significant increase in the GSH and CAT levels in CM-administered DM rats. We suggest that CM reduced the oxidative stress in STZ-induced testicular function. Therefore, CM can improve sperm concentration, motility, and viability. Moreover, another study reported that CM administration significantly increases the sperm count and motility in BPA-induced damage to the reproductive organs in rats [20]. Another study showed that diet supplemented with 1% and 5% CM mycelium could significantly enhance the sperm concentration in rats [16].

In agreement with the previous findings [1,9,15,27], our results showed that STZ-induced DM led to a significant reduction in the serum testosterone level and suppressed mating behavior. However, CM administration enhanced the testosterone level and sexual behavior in STZ-induced diabetic rats. Interestingly, CM supplementation promoted sexual behavior in not only diabetic rats but also healthy rats. The previous studies have reported that *C. sinensis* improved libido in men and women [55,56]. In corroboration with our findings, studies have reported that CM supplementation in male rats increased the serum testosterone level [20]. Particularly, a study indicated that serum testosterone and estrogen levels significantly increased in 2 and 6 weeks after CM supplementation in male rats [16]. In male reproductive function, testosterone plays an important role in the development of reproductive organs, spermatogenesis, and sexual behavior. From the observations of our experiment, it was indicated that an increased serum testosterone level after CM treatment may be responsible for the increase in sexual desire and libido. The previous studies suggested that CM could stimulate testosterone production by cordycepin, as adenosine dialogues. It stimulates cAMP-PKA-Star pathway and steroidogenesis in the Leydig cell of a mouse [57]. However, another study reported that cordycepin stimulated the intracellular phospholipase C/protein kinase C (PLC/PKC and MAPK) signal transduction pathway to induce steroidogenesis of the MA-10 Leydig tumor cell [58]. Another study reported that polysaccharide and/or glycoprotein in *C. sinensis* might have similar structure to the luteinizing hormone (LH) and possesses properties to stimulate the production of testosterone in Leydig cell through the LH receptor [59,60]. Our results showed that CM ameliorates DM-induced reproductive dysfunction in rats.

## Conclusion

Our results indicate that CM effectively improves reproductive function through enhanced testosterone levels and ameliorates oxidative stress and in turn the sexual behavior. Further study is required to determine the role of CM in the regulation of steroidogenesis and sexual behavior in healthy and diabetic rats. CM can be a potential drug supplement to reduce reproductive impairment resulting from DM.

## Authors’ Contributions

TVN, KP, and KS: Data collection, statistical analysis, and drafted the manuscript. PC: HPLC analysis, statistical analysis, and revised the manuscript. WF: Compiled the research idea and designed the main framework, revised the manuscript. All authors have read and approved the final manuscript.
